# Aortic valve Replacement compared to Transcatheter Implant and its relationship with COgnitive Impairment (ARTICO) evaluated with neuropsychological and advanced neuroimaging: a longitudinal cohort study

**DOI:** 10.1186/s12883-023-03362-9

**Published:** 2023-08-23

**Authors:** Meritxell Gomis, Claudio Fernández, Rosalia Dacosta-Aguayo, Xavi Carrillo, Silvia Martínez, Christian Muñoz Guijosa, Elisabet Berastegui, Antonio García Valentín, Josep Puig, Eva Bernal, Anna Ramos, Cynthia Cáceres

**Affiliations:** 1 Department of Neurosciences, Servei de Neurologia, Unitat d’Ictus, Hospital Universitari Germans Trias i Pujol, Universitat Auntònoma de Barcelona, Barcelona, Badalona Spain; 2 Servei de Cirurgia Cardíaca, Hospital Universitari Germans Trias i Pujol, Universitat Auntònoma de Barcelona, Barcelona, Badalona Spain; 3 Institut Universitari d’Investigació en Atenció Primària Jordi Gol (IDIAP Jordi Gol), Mataró, Spain Department of Clinical Psychology and Psychobiology, Institut Germans Trias i Pujol (IGTP) Unitat de Suport a la Recerca Metropolitana Nord, University of Barcelona, Barcelona, Spain; 4 Àrea del Cor, Servei de Cardiologia i de la Unitat d’Hemodinàmica i Cardiologia Intervencionista, Hospital Universitari Germans Trias i Pujol, Universitat Auntònoma de Barcelona, Barcelona, Badalona Spain; 5 Department of Neurosciences, Servei de Neurologia, Unitat de Neuropsicologia, Hospital Universitari Germans Trias i Pujol, Universitat Auntònoma de Barcelona, Barcelona, Badalona Spain; 6 Servicio de Cirugía Cardíaca, Hospital General Universitario de Alicante, Valencia, Spain; 7 Centre de Medicina Comparativa i Bioimatge de Catalunya, Institut de Recerca Germans Trias i Pujol, Barcelona, Badalona Spain

**Keywords:** Cognitive decline, Aortic valve replacement, Transcatheter implant, Advanced neuroimaging, Neuropsychological evaluation, Stroke

## Abstract

**Background:**

Aortic stenosis is the most common valvulopathy in Western countries. The treatment of choice had been surgery aortic valve replacement (SAVR), but the improvement in endovascular approaches as transcatheter aortic valve implantation (TAVI), initially reserved for patients with very high surgical risk, has been extended to high and intermediate, and recently also to low-risk patients. Stroke and vascular cognitive impairment are the most important complications. It is not entirely clear which technique is best to avoid these complications as well as their impact. Our goal is to evaluate changes in cognitive performance in the early (1-month) and late (1-year) postoperative period in patients undergoing SAVR or TAVI, by extensive neuropsychological study (NRP) and advanced Magnetic Resonance Imaging (MRI).

Specifically, to compare early and late cognitive changes after the intervention between both groups, the occurrence of stroke during follow-up and to compare the appearance of silent vascular lesions and changes in brain activity and functional connectivity with functional MRI during follow-up between both groups.

**Methods/design:**

Prospective longitudinal cohort study. A non-selected representative sample of 80 subjects, 40 SAVR and 40 TAVI to obtain a final sample of 36 eligible subjects in each group, ranging from 70 to 85 years old, with indication for aortic replacement and intermediate or high surgical risk will be studied. At baseline, within one month before the treatment, all individuals will undergo an extensive NRP and advanced MRI study. These studies will also be performed 1-month and 1-year after treatment, to assess the appearance of new vascular lesions, as well as changes in cognitive performance with respect to baseline.

**Discussion:**

This study aims to evaluate changes in cognitive performance as well as both clinical and silent vascular events occurring in the early (1-month) and late (1-year) periods after SAVR and TAVI. We will also analyze the correlation between neuropsychological and neuroimaging approaches in order to evaluate cognition. Therefore, it may provide high-quality data of cognitive changes and vascular events for both techniques, and be useful to tailor interventions to individual characteristics and ultimately aiding in decision-making.

**Trial registration:**

This study is register in Clinicaltrials.gov (NCT05235529) on 11^th^ February 2022.

## Background

Degenerative aortic stenosis (AS) is the most common valve heart disease among older patients in developed countries, with an exponential increase in prevalence with age. A meta-analysis conducted in those countries found a population prevalence of AS and severe AS in those aged 75 years and older of 12.4% and 3.4% respectively [1, 2].

Surgical aortic valve replacement (SAVR) has been the treatment of choice for patients with severe AS, but it is not appropriate for high-risk patients. In this scenario, over the past decade, several randomized controlled trials and observational studies have established the non-inferiority and even superiority of transcatheter aortic valve implantation (TAVI) compared with SAVR in high-risk patients or those considered inoperable [3–6].

Improvement in the learning curves of the endovascular technique, in the devices, and in the access routes have led to the indication of TAVI in intermediate-risk patients [7–9]. For this reason, nowadays-international guidelines have recommended the use of TAVI in inoperable and high-risk patients (Class I) and in intermediate-risk patients (Class IIa) [10–12].

Previous results and the minimally invasive technique of the TAVI procedure have encouraged investigations, as the recently published PARTNER 3 and Evolut Low-Risk trials [13, 14], showing non-inferiority and even superiority of TAVI compared with SAVR for some of the outcomes in low-risk patients.

Mortality and postoperative complications in patients undergoing aortic valve replacement are variable. In this study, we will focus on the most important and potentially disabling cerebral vascular events and vascular cognitive impairment (VCI).

In 2010 the PARTNER randomized trial showed a significant increase in stroke in the TAVI group compared with SAVR (5.5% and 2.4%) [4]. Two meta-analyses published in 2013 [15, 16] did not show significant differences (3.5 vs. 2.8% and 2.6 vs. 2.3%). Although the rate of stroke may have decreased marginally as TAVI has improved, questions continue to arise about the importance of microembolisms or silent cerebral infarcts that may cause changes in cognition. TAVI is associated with a high incidence (up to 84%) of silent cerebral embolism as detected by diffusion-weighted MRI (DW MRI) [17], more frequent than that following aortic valve surgery [18]. However, a clear correlation between the number or volume of vascular lesions and cognitive impairment has not been demonstrated.

Globally, cognitive impairment after cardiac surgery is one of the most frequent complications, particularly in aortic valve replacement (AVR). The definite cause of VCI after cardiac surgery is not known, but it could be attributed to cerebral embolic lesions due to gas particles, fat particles from the vascular wall, and calcium particles released as a result of manipulation [19]. In addition, the SENTINEL study showed that patients undergoing TAVI had a higher prevalence of cognitive impairment prior to TAVI, with a relationship between baseline cognitive function and burden of vascular lesions attributable to chronic cerebrovascular disease [20]. Therefore, VCI seen in patients with severe AS undergoing either endovascular or surgical treatment is multifactorial and involves pre-treatment as well as procedure-related and post-treatment factors. These findings underscore the importance of pre-intervention cognitive and neuroimaging tests in studies whose objective is to investigate post-surgical cognitive changes in patients with cardiovascular disease. A recent review analyzing data from studies of neurocognitive status after AVR and the differences between surgical and endovascular approaches show more consistent and favorable neurocognitive outcomes for TAVI patients, as the latest SAVR and TAVI trials have demonstrated [13, 14]. In addition, cerebral embolic protection devices offer the prospect of further improvement [21]. Nevertheless, there are several limitations to recent studies.

First, some studies use a single neuropsychological test as the primary variable for assessing changes in cognition [22–25]. In addition, only a few studies assess cognitive function in the long term [26, 27], which implies at least 6 months after the procedure. Furthermore, the definition of cognitive decline has varied from study to study, often defined simply by a decrease in mean scores in neurocognitive tests [28], and they differ with regard to the neuropsychological tests and batteries used, making it difficult for the comparability of results. Finally, it is important to consider other factors such as low educational level and medical comorbidities such as preexisting cardiovascular disease since they have been found to be risk factors for VCI [29].

Advanced imaging studies, such as resting-state MRI (rs_MRI), is used in brain mapping to assess regional interactions that occur in a negative task. Several resting-state conditions have been identified in the brain, one of which is the Default Mode Network (DMN), which is more active during the rest period than during task execution. The Salience Network (SN) is key because it regulates the activation of the Central Executive Network (CEN) while deactivating the DMN during the execution of a task. In the case of cognition, it is interesting to study the integration of these different neural networks. Interestingly, a study on VCI found differences in rs_RNM between subjects with and without cognitive impairment, which also correlate with neuropsychological tests such as the Montreal Cognitive Assessment (MoCA) [30].

In this context of uncertainty regarding the clinical importance of stroke and VCI in patients that undergo SAVR and TAVI, we designed a prospective study called ARTiCO (Aortic valve Replacement compared to Transcatheter implant and its relationship with Cognitive impairment). The specific objectives are;(1) to compare the early and late cognitive changes after the intervention in both groups;(2) to compare the occurrence of stroke during follow-up in both groups;(3) to quantify and compare the appearance of silent vascular lesions in the MRI at follow-up in both groups;(4) to study with functional MRI, changes in brain activity and functional connectivity and to correlate them with NRP functioning.

Therefore, the study aims to provide relevant information to determine whether cognitive dysfunction appears, persists, increases or even decreases and whether it does so differently between the SAVR and TAVI groups. We also will study the clinical and silent incidence of cerebral vascular disease between both techniques. Finally, we will analyze the correlation between neuropsychological and neuroimaging approaches in order to evaluate cognition.

The final goal of the study is to gather evidence that will help the Heart Team in decision-making processes regarding which treatment to apply to patients with the indication of AVR. This article describes the ARTiCO study protocol.

## Methods/design

### Aim, design and setting of the study

This is a prospective longitudinal cohort study that will include eighty non-selected consecutive subjects ranging from 70 to 85 years old with severe AS with an indication for AVR and an intermediate or high surgical risk. The participants will be evaluated by the Heart Team of a Comprehensive Center (Germans Trias i Pujol Hospital) that will assign them to SAVR, TAVI, or conservative treatment. Despite the limitation of this is not a randomized study; only those subjects who are suitable for both interventions will be included (and in many cases, the decision to undertake one treatment or another will made on the basis of availability for one or another technique). Subjects will be evaluated in three time periods: at baseline, within 1 month prior to intervention, 1 month after the intervention (early follow-up), and 1 year after the intervention (late follow-up). Throughout these periods, all study subjects will concomitantly undergo an extensive clinical examination, including neurological examination, neuropsychological evaluation, and advanced neuroimaging protocol to determine clinical and silent stroke events and cognitive status. In addition, we will study the correlation between neuropsychological and neuroimaging findings.

### Subject selection

This study will be carried out at the Germans Trias I Pujol University Hospital, a public health tertiary center of Catalonia. The advanced neuroimaging will be performed in the Comparative Medicine and Bioimage Center of Catalonia (CMCiB), a referent technological image equipment of the Germans Trias I Pujol Research Institute. The protocol has been approved by the Ethics Committee of our Institution.

The Heart Team of our hospital evaluates patients with a diagnosis of severe AS from an area of influence of approximately 600,000 inhabitants from both a rural and urban population. About 12,000 meet the diagnosis of severe AS, and 360 is the number of patients per year treated in our hospital with SAVR or TAVI. Taking into account that approximately 50% of these 360 patients underwent non-elective treatment, the number of screening candidates for the ARTiCO study screening is about 180 patients per year.

**Fig. 1:**
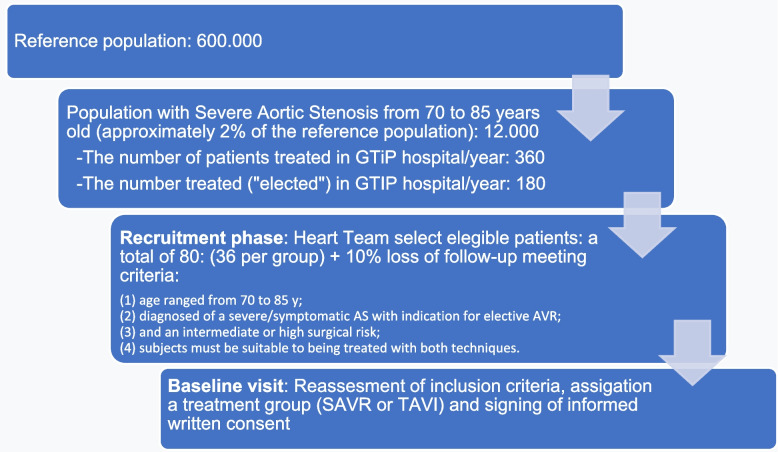
ARTiCO study sample selection. The diagram shows the sample selection in ARTiCO study from the reference population. SAVR: surgical aortic valve replacement; TAVI: transcathether aortic valve implantation.

Once patients are assigned to a treatment, the Cardiac Surgeon in the case of SAVR or the Cardiologist in the case of TAVI will verify that they meet all of the following inclusion criteria. The inclusion criteria are: (1) age range from 70 to 85 years old; (2) diagnosis of a severe/symptomatic AS with an indication for elective AVR; (3) an intermediate or high surgical risk evaluated by the EuroScore II (3-10%); and (4) subjects must be eligible to be treated with both techniques. Subjects will be excluded if they have: (1) contraindications for MRI, (2) severe renal failure, (3) severe disability or previous chronic neurologic or/psychiatric disease, (4) diagnosis of dementia or Mini-Mental State Examination (MMSE) (MEC de Lobo Spanish version) <19/35 corrected for age and education [31], (5) those with previous cardiac surgery, (6) patients with severe preoperative comorbidities that may difficult a 1-year complete follow-up, (7) aortic surgery concomitant to that of AVR or other than coronary revascularization. Excluded patients will be registered for quality control in the selection of study patients. All subjects who meet all the inclusion criteria and none of the exclusion criteria will be invited to participate in the study. If they accept, they will sign the informed consent form. (Figure 1)

The sample size calculation will be based on the main study variable, the Global Cognitive Impairment Index (GCII), which has the Impairment Index or Global Cognitive Impairment Index, as a standardized normal distribution. Setting an alpha error of 5% and a beta error of 20% (80% statistical power) for a two-sided analysis and an effect size to observe defined as a minimal difference in z score of 0.67. Applying these criteria, we obtain an estimate of 36 patients per group. Taking into account a 10% loss to follow-up, we need a total sample of 80 subjects.

### Baseline procedures and data collection at the initial visit conducted within 1 month prior to the intervention: (Figure 2)

**Fig. 2:**
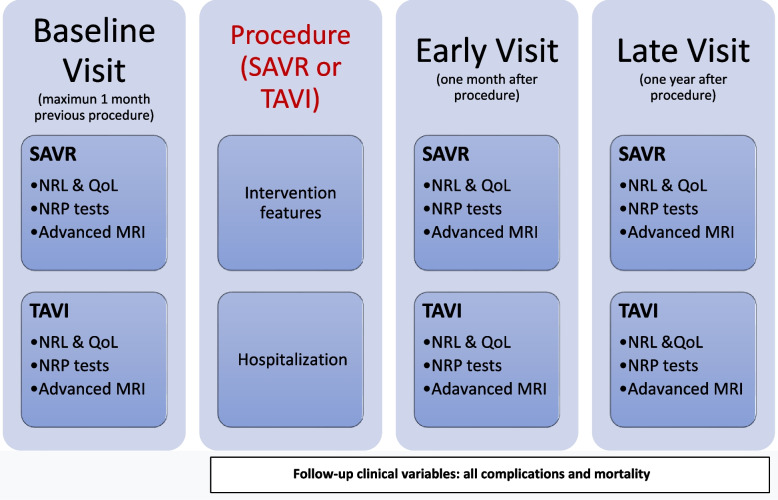
ARTiCO study flow chart. The diagram shows baseline and follow-up procedures in ARTiCO study. SAVR: surgical aortic valve replacement; TAVI: transcatheter aortic valve implantation, NRL: neurological evaluation; NRP: neuropsychological evaluation; MRI: magnetic resonance imaging, QoL: quality of life tests.

1. Signing of informed written consent
2. Clinical data will be collected in a questionnaire specifically designed for this study:

- o Sociodemographic variables: age, sex, education (years and level of education), hand dominance
- o Anthropometric variables: height, weight.
- o Vascular risk factors: smoking habit (never, current, former), alcohol intake, physical activity level (sedentary, home-activity, outdoor activity and grade), history of hypertension, diabetes mellitus, dyslipidemia, and arrhythmia (atrial fibrillation or another type).
- o Current drug intake (platelet inhibitors, anticoagulants, lipid-lowering drugs, antihypertensives, hypoglycemic agents, antiarrhythmics, antidepressants).
- o Vascular events presented prior to inclusion in the study: angina, myocardial infarction, intermittent claudication, transient ischemic attack, and stroke.
- o Heart Failure: Using the New York Heart Association classification (From class I to IV)
- o Renal function before treatment (creatinine level and glomerular filtrate)
- o Total score in EuroScore II  [32]

3 Neurological evaluation
  A complete neurological evaluation will be carried out by a neurologist specialized in cerebrovascular diseases. It will include; a complete anamnesis and review of the subject’s clinical history, including data from neuroimaging studies to confirm the existence of previous cerebrovascular pathology in any of its manifestations (Type 1: overt Central Nervous System (CNS) injury; Type 2: covert CNS injury, and Type 3: neurologic dysfunction without CNS injury) following the Neurologic Academic Research Consortium  (NeuroARC) recommendations [33]. Likewise, a neurological examination will be carried out using the National Institutes of Health Stroke Scale (NIHSS) [34] to detect neurological deficit attributable to cerebrovascular lesions, with a score range of 0 to 42, with 0 indicating absence of focal neurological damage. Functional dependence will be assessed with the modified Rankin scale (mRS) ranging from 0 to 6, where 0 indicates functional independence and 6 death [35]. In addition, depressive symptoms will be assessed with the Geriatric Depression Scale (GDS), with scores higher than five indicating probable depression [36], and cognition will be assessed by the Informant Questionnaire On Cognitive Decline in the Elderly (IQCODE) test with scores higher than 57 indicative of probable cognitive decline [37]. The same stroke neurologist will assess all cerebrovascular events that subjects may experience during follow-up.
4 Neuropsychological assessment:

- Acquisition, analysis of neuropsychological variables:
  - Neuropsychological Tests:
    The same clinical neuropsychologist will conduct the NPS evaluation at the three evaluation times, and the neuropsychologist will be blind to the patient’s treatment group.
    The neuropsychological battery will last approximately one and a half hours, and it will consist of tests sensitive to vascular cognitive impairment (VCI). Neuropsychological tasks will be categorized into four cognitive domains:
    - Attention and Psychomotor speed will be measured by the Digit Span forward subtest of the Wechsler Adult Intelligence Scale (WAIS-III) [38], the Symbol Digit Modality Test (SDMT) [39], the Symbol Search subtest of the WAIS-III [38], the Grooved Pegboard dominant hand [40] and the Trail Making Test part-A (TMTA) [39, 41].
    - Verbal and visual memory will be evaluated with the Rey Auditory Verbal Learning Test (RAVLT) 15-word verbal learning test (RAVLT) [42] and the delayed memory recall of Rey's Complex Figure (RCF) [43].
    - Executive functions will be measured by the Digit Span backward subtest WAIS-III [38], the Trail Making Test part-B (TMTB) [39, 41], and the Phonemic Verbal Fluency test (letter M) [44].
    - Visuoconstructive and Visuospatial functions included the copy of Rey’s Complex Figure (RCF) [43] and Block design subtest of the WAIS-III [38].

Also general cognitive functioning will be evaluated before the neuropsychological battery using the MMSE and MoCA tests [31, 45].

The order in the administration of tests will be constant to avoid variability between subjects due to fatigue.

To control for type I errors associated with multiple comparisons and to summarize scores obtained from the wide range of tests used, we will compute a Global Cognitive impairment Index (GCII) as the primary outcome. To this end, all measures will be first transformed to standardized z scores using age and education level corrected norms. The GCII will be computed by averaging all z scores from the different measures and also separately for each cognitive dimension. The final GCII will be a z score with a mean of 0 and an SD of 1 with lower or negative scores reflecting a poorer performance.

In addition, all patients will be classified into one of three groups according to their performance on the neuropsychological battery: a group with no cognitive impairment, a group with mild cognitive impairment (MCI) and a group with moderate cognitive impairment.

MCI will be defined when the results of one or more cognitive domains are 1.5 SD below the mean. Moderate cognitive impairment will be considered when the results of one or more cognitive domains are 2 SD below the mean [46].

5 Advanced Neuroimaging study protocol:

- Data acquisition

Assessments will be carried out at the CMCiB with an MRI (Cannon MRT-3020 Vantage Galan 3T) dedicated to biomedical research. We will use a 32-channel phased-array head coil with foam padding and headphones to restrict head motion and suppress scanner noise. The MRI protocol will include a set of magnetization-prepared rapid gradient echo (MP-RAGE) T1-weighted images (repetition time [TR]: 8.4 ms; echo time [TE]: 2.7 ms; field of view: 256 mm; flip angle: 9º; and voxel size: 1×1×1 mm3). T2*- weighted images will be acquired using a multi-echo sequence with the following acquisition parameters: repetition time [TR]: 845.8 ms; with the following echo times [TE]: 6.800, 13.600, 20.400, and 27.200 ms; field of view: 245 mm; flip angle: 20º, and voxel size: 0.80×0.80×3 mm3. Fluid Attenuated Inversion Recovery (FLAIR) image will acquire with the following acquisition parameters: repetition time [TR]: 7000 ms; echo time [TE]: 445.5 ms; inversion time [TI]: 2200 ms; field of view: 256 mm; flip angle: 90º; and voxel size: 1×1×1 mm3. DWI will acquire in 48 non-collinear diffusion directions, with a b-value of 1.000 s/mm2, with the following echo planar acquisition protocol: [TR]: 12447 ms; [TE]: 81 ms; field of view: 240×256 mm; flip angle: 90º; and voxel size: 2×2×2 mm3; phase-encoding direction: PA. Two images with a value of 0 s/mm2 and opposite phase-encoding directions (AP and PA) will also be acquired. Intravoxel Incoherent Motion images (IVIM-DWI) will also be acquired with a single-shot spin-echo echo-planar imaging sequence using the following acquisition protocol: [TR]: 6680 ms; [TE]: 90 ms; field of view: 240 mm; flip angle: 90º; and voxel size: 1.5×1.5×5 mm3 with six b-values (50 sec/mm^2^, 100 sec/mm^2^, 300 sec/mm^2^, 600 sec/mm^2^, 1200 sec/mm^2^, and 2400 sec/mm^2^ with three orthogonal acquisition directions for each b-value. Resting-state blood oxygen level-dependent data will be acquired using an echo-planar imaging sequence (repetition time = 2.25 s; echo time = 25 ms; flip angle = 90°; in-plane spatial resolution = 3 × 3 mm2; field of view = 240 × 240 mm2; slice thickness = 3 mm; number of slices = 37; number of volumes = 256; acquisition time = 8:32 min). Participants will be instructed to lie still with their eyes closed but remain awake.

- Data analysis

Cortical thickness and volume analysis will be carried out with Freesurfer 7.3.1 (http://www.surfer.nmr.mgh.harvard.edu/), and the pre-processing and analysis of the DWI and resting-state fMRI images FSL 6.0.5.2 (FMRIB's Software Library, http://www.fmrib.ox.ac.uk/fsl/).

DWI pre-processing included motion and eddy current correction using FSL’s Topup and Eddy Correct Tool using the FMRIB Diffusion Toolbox (FDT) (Analysis Group, FMRIB, Oxford, UK) [47]. In order to eliminate spurious voxels, skull stripping of the T2 weighted b = 0 volume was achieved using FSL’s Brain Extraction Tool (BET) and will be used as a brain-mask for all other diffusion maps. FDT will be used for the tensor modeling of the diffusion parameters to produce DTI data. Microstructural maps of axial (AD), radial (RD), and mean (MD) diffusivity and fractional anisotropy (FA) will be entered into group analysis using Tract Based Spatial Statistics - TBSS [48]. All subjects' FA data will be aligned into a common space using the nonlinear registration tool FNIRT [49, 50], which uses a b-spline representation of the registration warp field [51, 52], resulting in all images transformed into 1 mm isotropic, MNI152 standard space. Next, all participants’ FA volumes will average, and a mean FA skeleton will be created from all voxels with an FA threshold = 0.2 to reduce the inclusion of voxels that are likely composed of multiple tissue types or fiber orientations. Each participant's aligned, standard space FA maps will be then projected onto this skeleton to create a 4D skeletonized volume (3D skeletal volume × number of subjects) which will then fed into voxel-wise group statistics. Other diffusion-derived data (AD, RD, and MD) projections on the TBSS skeleton will also be calculated for each subject. The spatial normalization transformations computed for the FA maps will be applied to these maps to achieve their nonlinear registration, which will be projected on the TBSS skeleton. The resulting 4D volumes will also be used for voxel-wise cross-subject statistics. For the analysis of structural connectivity, the probabilistic tractography analysis (Probtrackx2) will be used.

**Fig. 3:**
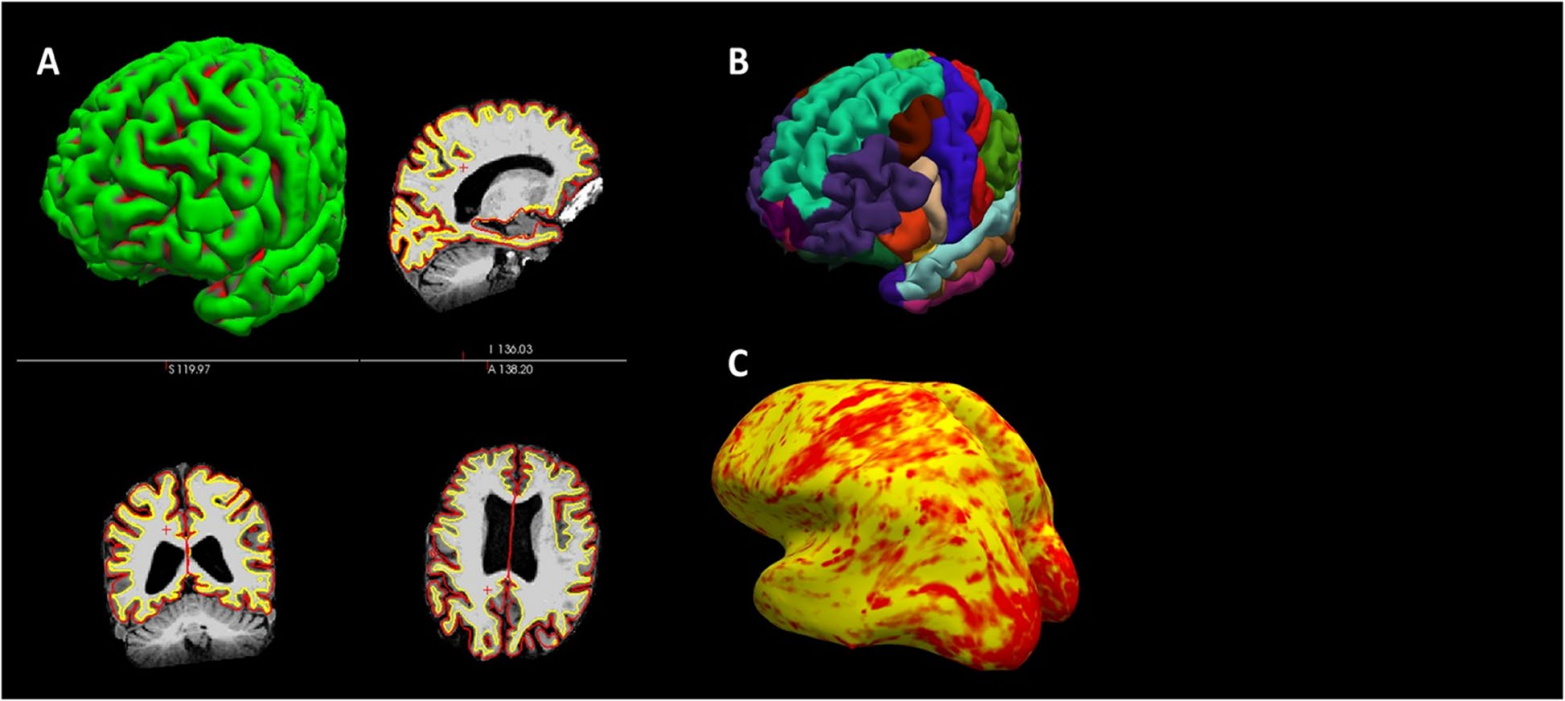
Demonstrates the FreeSurfer pipeline, including tissue segmentation **A**, parcellation based on the Desikan-Killiany Atlas **B**, and estimation of the cortical thickness (**C**) of the structural, T1-weighted acquisition.

**Fig. 4:**
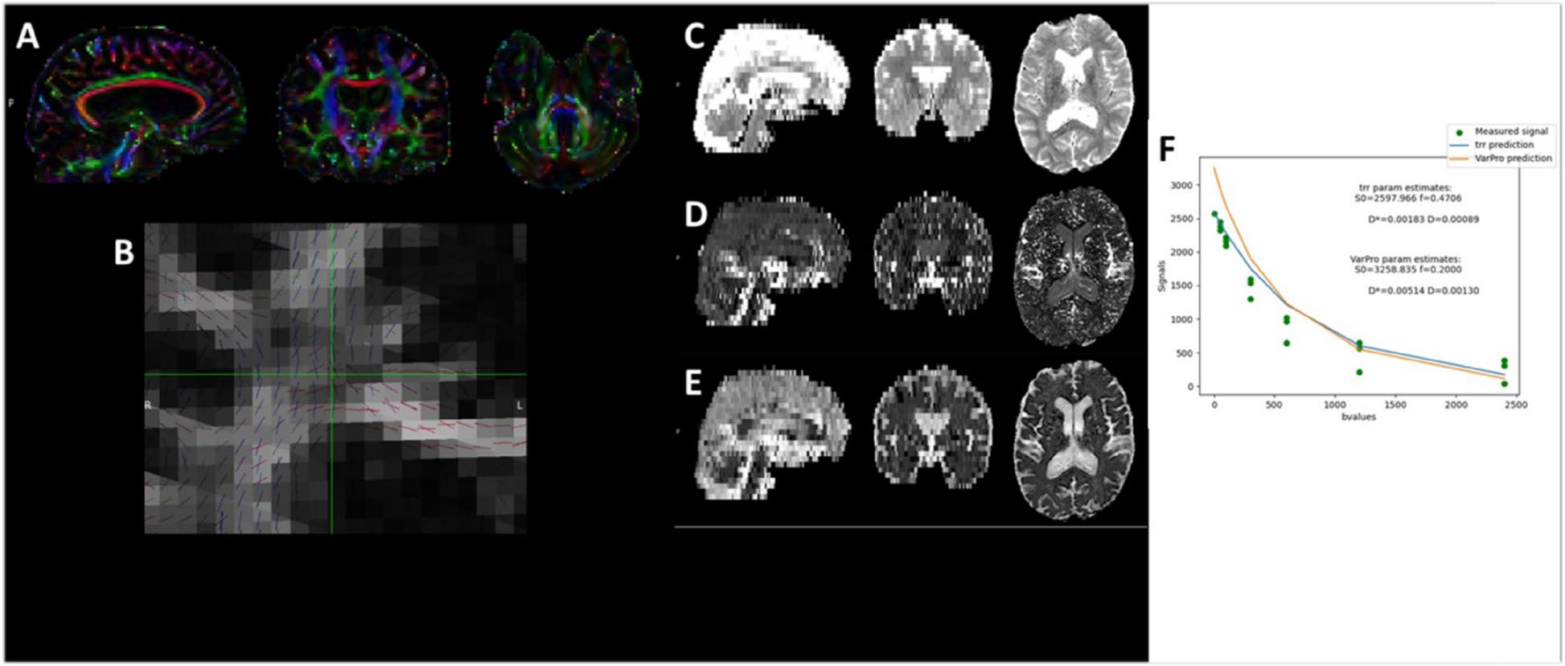
Processing the diffusion-weighted data. **A** and **B** demonstrates the pipeline for processing the diffusion-weighted data, including estimating the primary direction of diffusion (**A**), and co-occurring fibers at the crossing three major tracts (**B**), using FSL’s DTIFIT and BedpostX. **C**, **D** and **E** shows the parameter estimates **D** (**C**), D* (**D**), and f (**E**) of the voxel-wise estimation of Intravoxel Incoherent Motion, while F shows the fitting of the model on a typical voxel.

**Fig. 5:**
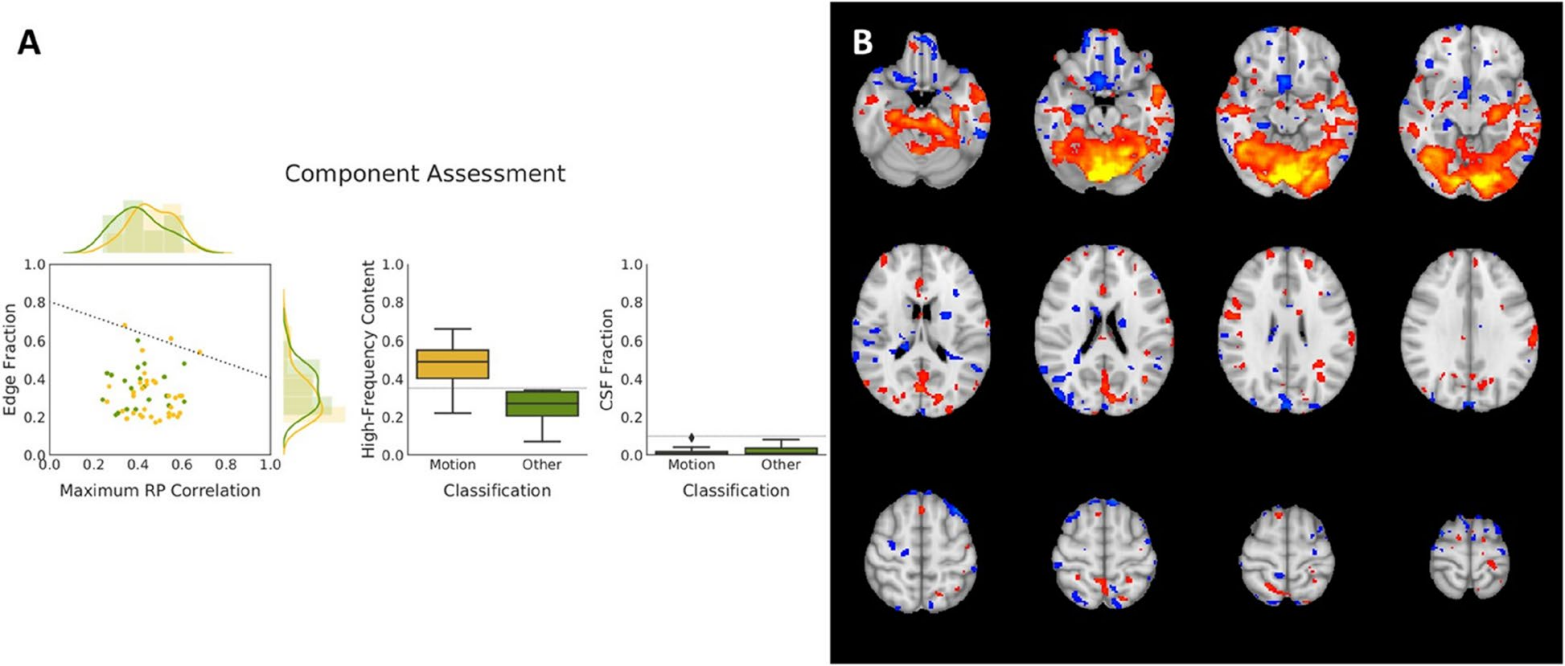
Demonstrates the ICA-AROMA pipeline for cleaning the resting-state fMRI data, including the component assessment (**A**) and a component clearly showing motion artefacts (**B**).

The acquired IVIM-DWI images will be processed using DIPY 1.6.0.[53] IVIM-D and IVIM-f maps will be calculated in native space using the “2-stage Trust-Region Reflective based NLLS fitting method” (TRR) fitting method [54]. The analysis of the resting-state fMRI data will be conducted using probabilistic Independent Component Analysis as implemented in FSL’s MELODIC tool. Data preprocessing will consist of the removal of the first 5 volumes to ensure saturation and adaptation of the subjects to the environment leaving 251 volumes for further analysis, removal of non-brain structures using Brain Extraction Tool, motion correction using MCFLIRT, high-pass filtering with a frequency cut-off at 100 s, spatial smoothing using a Gaussian kernel of full-width half-maximum of 5 mm, intensity normalization, and non-linear registration to the MNI152 standard template. We will discard components representing known artifacts, such as motion, high-frequency noise, or venous pulsation [55, 56] components not located mainly in gray matter, and components not resulting in compact clusters [57] using ICA-AROMA [49]. (Figures 3, 4 and 5)

### Procedures and data collection during the intervention and hospitalization period

Intraoperative features:

- Extracorporeal circulation time, coronary ischemia time, and orotracheal intubation
- Bleeding from drains
- Transfusion of blood products
- Types of prosthesis and approach: in SAVR (Percebal S (LlivaNova PCL) which is a suture-less bio-prosthesis, and in TAVI (Sapien 3 (Edwards Lifesciences).
- Total of intubation hours and need for tracheostomy

During hospital admission

Definite morbidity at discharge:

- Stay in the ICU for>48 hours, in the ward for more than 10 days
- Respiratory, renal (RIFLE criteria), infectious, cardiac rhythm complications (endo-cavitary pacemaker implant)
- Neurological complications: stroke, transient ischemic attack, episodes of disorientation and seizures
- Degree of dependency
- Readmissions (in the critical/semi-critical unit)
- Valvular hemodynamics (echocardiographic data)

Definite Mortality at discharge:

- Immediate: <72 hours post-procedure
- Hospital: during hospitalization and/or first 30 days post-procedure

Follow-up clinical variables: All complications and mortality at 6 months and one year will be collected, as well as valve hemodynamics and functional class in both groups.

### Early visit procedures of the study protocol, planned to be performed approximately one month after the operation:

1. Neurological Evaluation
  The stroke neurologist will question the patient to rule out the existence of cerebral vascular events from the baseline visit and, if detected, will carry out an etiological study thereof. The events will be classified following the (NeuroARC) recommendations. Regardless of the existence of cerebral vascular events, the neurologist will perform the neurological examination with the NIHSS and will administer themRS, as well as the GDS and the SS-IQCODE.

2 Neuropsychological assessment:
  The same neuropsychologist who administer the tests at the baseline visit will administer them again at the monthly visit. I would like to draw attention to the fact that the tests will be reviewed by another neuropsychologist of the study to avoid errors before transferring these results to the electronic data notebook. This procedure will be performed at all the study visits in which the neuropsychological study will be administered.

3 Advanced Neuroimaging study protocol:
  The same study protocol will be administered at this visit. Two blinded investigators to the study group assignment will read the images.

### Late visit procedures of the study protocol, planned to be performed approximately one year after the operation:

1. Neurological Evaluation
  In the final visit of the study, the vascular neurologist will ensure through questioning and review of the medical history that the patient has not suffered any cerebral vascular event, and if so, it will carry out an etiological study of the same. The events will be classified following the NeuroARC recommendations. Regardless of the existence of cerebral vascular events, the neurologist will perform the neurological examination with the NIHSS and will administer themRS , as well as the GDS and the SS-IQCODE.

2 Neuropsychological assessment:
  The same neuropsychologist who administered the tests at the baseline and early visit will administer them again at the year visit.

3 Advanced Neuroimaging study protocol:
  The same study protocol performed at the baseline, and early visits will be administered at this visit. As in previous visits, two investigators blinded to the study group assignment will read the images.

### ARTiCO clinical and image database

Clinical data will be recorded in case report forms (CRF). The data from the neuropsychological and neuroimaging studies will be collected in different CRFs that the one that stores the clinical data to maintain blindness. The images obtained in advanced MRI studies will be stored on specific CDs to be analyzed afterward. A prospective electronic database with clinical, neurological, neuropsychological, and neuroimaging variables will be created, including all study visits.

### Statistical considerations

The sample size calculation will be based on the main variable of the study, Impairment Index or Global Cognitive Impairment Index, which will be handled as a standardized normal distribution (mean=0 and SD=1). The way to transform the values of every subject to a standardized value is using the formula (value-mean)/SD. With this approach, we can estimate the difference between groups as a difference in the standardized normal distribution (z score), avoiding to approximate or guess values of SD that we do not previously know.

Establishing an alpha error of 5% and a beta error of 20% (80% statistical power), for a two-sided analysis and an effect size to observe defined as a minimal difference in z score of 0.67. Applying these criteria, an estimate of 36 patients per group is obtained. Assuming a loss of follow-up of 10%, we aimed to recruit 40 patients per group. The statistical package used for sample calculations was Stata 14.2 (StataCorp LLC, College Station, Texas, USA).

A descriptive analysis of the sample will be carried out based on the characteristics of each variable. Continuous variables will be expressed as mean and 95% confidence interval, or median and interquartile range for each of the study groups, choosing the most appropriate description according to the result of the Shapiro-Wilk normality test. Categorical variables will be expressed with their absolute frequency (count) and relative frequency (as a percentage).Data cleansing and exploration of missing values will be carried out. Missing data greater than 10% will be considered excessive, deciding whether to maintain the variable in the study according to its importance in the literature and in the study design. For missing data recognized as excessive in clinically important variables, the dataset will be completed using multiple imputation techniques. Student t-test will be preferred for the crude comparative analysis between groups as a bilateral parametric test that will allow us to find differences in both directions of the hypothesis, given the heterogeneity of the existing literature. Equality of variances will be tested using the Levene test to adjust the standard error in the comparison. Sample size should ensure the conditions of application of parametric tests.The statistical adjustment of confounding factors and interaction of predictors will be carried outby building an explanatory model using multivariate linear regression, thus obtaining the coefficient corresponding to the main variable adjusted by the rest of potentially confounding or effect-modifying variables. First, a univariate screening of variables will be carried out, introducing in the model those which present confounding criteria based on the Pearson correlation test. Subsequently, first-order interactions will be explored based on statistical significance tests. The adjustment of confounding factors will be carried out based on relevant modification criteria (larger than 10%) of the model coefficients, according to the methodology described by Kleinbaum et al [58], and applying the principle of parsimony. The search for independent predictors of neuropsychological dysfunction will be carried out using a predictive approach of hierarchical logistic regression with a stepwise backward methodology.An analysis of residuals and extreme values will be carried out to verify the robustness of the built model. For the rest of the secondary hypotheses, mean comparison techniques (Student's t-test with adapted standard error) and proportions (chi-square) will be used.

## Discussion

Despite recent improvements in endovascular treatment as an alternative to surgery in severe AS, even in low-risk subjects, neurocognitive outcomes after SAVR and TAVI remain unknown [59–61].

Cognitive impairment and stroke predict future functional decline, leading to reduced mobility, poor quality of life, and increased mortality [59, 60].. Recent studies that have included neurological adjudication and brain imaging have shown infarcts on MRI in up to 61% after SAVR, with clinical stroke in 17% [60]. On the other hand, previously reported high stroke rates after TAVI were probably due to increased verification of cerebrovascular events by stroke neurologists [13, 14]. Studies in patients undergoing TAVI have identified new brain lesions on MRI in 98% [62, 63]. In this line, the NeuroARC is one of three consortia that have developed endpoint definitions for assessing vascular events after SAVR and TAVI to standardize neurological outcomes [33]. All of this reinforces the idea that new studies that seek to compare both techniques require a neurologist who is a stroke expert to assess all clinical events and evaluating MRI images.

Regarding neurocognition, data from several trials showed that patients had a more significant decrease in MMSE score after SAVR compared with TAVI [64, 65]. It is important to note that the post hoc analysis of the PARTNER-3 low-risk population demonstrated cognitive improvement at 30 days in all those with pre-existing impairment and sustained improvement in the TAVI group at 1-year follow-up [66]. On the contrary, in two meta-analyses assessing cognitive outcomes after TAVI, one found no significant change in peri-procedural cognitive performance, an improvement at 1-month, but no significant improvement at 6 months or final follow-up [59]. The other, which has been recently published, showed that pre-existing cognitive impairment was a significant risk factor for poorer outcomes after TAVI, indicating that these patients should be carefully considered before inclusion in this treatment [67]. For this reason, studies on the cognitive aspects of SAVR and TAVI should always include a baseline neuropsychological and neurological evaluation performed prior to and close to the time of the intervention. It is also important to evaluate cognition early following the intervention and, in the long term, at least 6 months and ideally 1 year after the procedure.

Probably one of the most critical points in the evaluation of cognitive impairment is the selection and design of the neuropsychological test battery. Studies differ with regard to the neuropsychological tests used, making it difficult to establish meaningful comparisons. Despite the profile of cognitive impairment after AVR is VCI, somestudies use only one or two screening tests, usually the MMSE [22, 23], which lacks adequate sensitivity to detect VCI. Other studies use the MoCA, which is more sensitive to VCI but still provides only a brief and limited measurement of cognitive function [24, 25]. Only a complete NRP battery that includes all the necessary domains may be able to detect cognitive impairment following the procedures, which might otherwise go undetected.. Some studies analyze the correlation between specific cognitive domains affected by VCI and subcortical vascular insult. For example, ischemic lesions in prefrontal-subcortical circuits have been associated with lower executive function, forgetfulness and changes in speech and emotion [60]. Some studies have explored these cognitive domains after TAVI, but the results were not homogeneous. A study that measured changes in delayed recall, working memory, verbal learning, and fluency immediately and three months after TAVI did not find significant differences [68]. Another study showed that visual attention and delayed recall improve at the early follow-up after TAVI [69]. On the other hand, a study that assessed changes in executive function, processing speech, and abstract reasoning demonstrated that about 25% of the subjects had an early decline in these domains, which remained at 40% at 1-year follow-up [70].

We would like to point out that together with the pre-existing cognitive impairment and the characteristics associated with the procedures, the other variable associated with cognitive changes in AVR is age. In older patients, the impact of cognitive impairment after AVR is particularly important because it may increase their morbidity and lower their quality of life. Interestingly, there is one study that demonstrated increasing older age as the only independent risk factor for cognitive impairment after TAVI, but not the other factors as cognitive status, prior stroke events, use of embolic protection devices, or silent cerebral lesions. [71].

Along with the neuropsychological study, which is the goal standard in the diagnosis of cognitive impairment, including VCI, neuroimaging with MRI is key to objectifying the lesions that are probably the origin of alterations in cognitive domains. In addition, functional MRI will allow us to study pathways and brain functions, as well as establish the correlation between the findings with the results of the neuropsychological study. The potential usefulness that can be obtained with the data from our study could mean using neuroimaging as an alternative to the neuropsychological study in those patients with cognitive alterations or with language alterations, such as aphasia, or of the senses such as hearing or sight, that do not allow the administration of all or some neuropsychological tests.

Strengths of this study are: 1). Although patients will not be randomized between SAVR and TAVI, they are eligible for inclusion in the study only if they are suitable for both techniques; therefore the groups will be comparable; 2). To the best of our knowledge, this is the first study that simultaneously uses an extensive neuropsychological study, with a battery designed to evaluate all domains likely to be affected in the VCI, and an advanced MRI study, with sequences to assess structural damage as well as a functional MRI to compare SAVR and TAVI in patients with severe aortic stenosis; 3). In addition, these studies will be carried out pre-treatment, in the early phase and in the late phase; 4). and will have the participation of a vascular neurologist who will evaluate the patients and the clinical events that they may present following the recommendations of the NeuroARC.

We hope that all the information obtained from ARTiCO study will contribute to a better understanding of the changes that occur in neurocognition after the two distinct types of treatment available for severe AS and therefore, that it helps Heart Teams in decision-making.

## Data Availability

We assure the availability of data and materials of the study.

## Abbreviations

AD: Axial diffusivity
ARTiCO: Aortic valve Replacement compared to Transcatheter implant and its relationship with COgnitive impairment
AS: aortic stenosis
AVR: aortic valve replacement
BET: Brain Extraction Tool
CEN: Central Executive Network
CMCiB: Comparative Medicine and Bioimage Center of Catalonia
CNS: Central Nervous System
CRF: Case report forms
DMN: Default Mode Network
DW MRI: diffusion-weighted MRI
FDT: FMRIB Diffusion Toolbox
FA: Fractional anisotropy
FLAR: Fluid Attenuated Inversion Recovery
GCII: Global Cognitive Impairment Index
GDS: Geriatric Depression Scale
IQCODE: Informant Questionnaire On Cognitive Decline in the Elderly
IVIM: Intravoxel Incoherent Motion images
MRI: Magnetic Resonance Imaging
MP-RAGE: Magnetization-prepared rapid gradient echo
MCI: Mild cognitive impairment
MD: Mean diffusivity
MMSE: Mini-Mental State Examination
MoCA: Montreal Cognitive Assessment
mRS: modified Rankin scale
NeuroARC: Neurologic Academic Research Consortium
NIHSS: National Institutes of Health Stroke Scale
NRP: neuropsychological study
NRL: Neurological evaluation
QoL: Quality of life tests
RD: Radial diffusivity
RAVLT: Rey Auditory Verbal Learning Test
RCF: Rey's Complex Figure
rs_MRI: resting-state MRI
SN: Salience Network
SDMT: Symbol Digit Modality Test
SAVR: surgery aortic valve replacement
TAVI: transcatheter aortic valve implantation
TMTA: Trail Making Test part-A
TMTB: Trail Making Test part-B
TRR: Trust region Reflective
VCI: vascular cognitive impairment
WAISS: Wechsler Adult Intelligence Scale
